# Utilisation and prognostic impact of angiography and primary percutaneous coronary intervention prior to intensive care admission for patients following out-of-hospital cardiac arrest

**DOI:** 10.1186/cc14503

**Published:** 2015-03-16

**Authors:** L Eveson, S Shrestha, M Davies, V Achan, M Peck

**Affiliations:** 1 Frimley Park Hospital, Frimley, UK

## Introduction

Our 700-bed hospital has a 24-7 cathlab service that routinely investigates patients with indications prior to ICU admission following out-of-hospital cardiac arrest (OHCA). Our aim was to compare ICU survivors and nonsurvivors and evaluate the utilisation and prognostic impact of angiography and primary percutaneous coronary intervention (PPCI) in this patient group.

## Methods

A retrospective analysis using Trust electronic databases (Symphony, WardWatcher, PICIS, PRISM) of all OHCA patients admitted to our ICU over 3 consecutive years between 1 November 2011 and 31 October 2014.

## Results

A total of 351 patients presented to our hospital following OHCA in this period, and of these 50% died in the ED, 37% were admitted to the ICU and 13% elsewhere. Of the 129 patients admitted to the ICU, median age was 66 (range 18 to 93), 71% were male, 68% had a shockable presenting rhythm, median ICNARC score was 31 (range 10 to 66), median ICU LOS was 3.75 (range 1 to 34 days) and ICU and hospital mortality were 50% and 60% respectively. ICU survivors were more likely to have had a shockable rhythm (89 vs. 55%), been to the cathlab (83 vs. 53%), received PPCI (43 vs. 26%) and TH (82 vs. 62%) and had lower median ICNARC scores (26 vs. 35) than nonsurvivors. Over the 3 consecutive years, pre-ICU cathlab and PPCI utilisation increased annually in ICU survivors (73 vs. 86 vs. 89% and 45 vs. 54 vs. 59% respectively) and nonsurvivors (40 vs. 38 vs. 50% and 20 vs. 27 vs. 31% respectively). However, our annual ICU and hospital mortality remained unchanged (46 vs. 51 vs. 51% and 60 vs. 57 vs. 62% respectively).

## Conclusion

ICU survivors were more likely to have had a shockable rhythm, been to the cathlab, received PPCI and TH and been less sick than nonsurvivors, but these may simply reflect selection and other biases. Any benefit these factors did confer to cardiac patients may have been offset by our liberal ICU admission policy to OHCAs with nonshockable rhythms. However, access to 24-7 PPCI may determine survival and we suggest that all OHCA patients should be taken directly to regional heart attack centres.

